# Comprehensive evaluation of aortic disease by in-vivo 4D flow MRI and 3D printing of patient-specific models: a feasibility study

**DOI:** 10.1186/1532-429X-18-S1-P365

**Published:** 2016-01-27

**Authors:** Rouzbeh R Ahmadian, Austin P Boyd, Alessandro M Scotti, Jeremy D Collins, James C Carr, Patrick M McCarthy, SC Malaisrie, Alex J Barker, Michael Markl

**Affiliations:** Radiology, Northwestern University, Chicago, IL USA

## Background

Aortic diseases are often associated with changes in vessel geometry and blood flow. Currently available tools provide limited regional information that do not fully capture these complex alterations. Studies have shown that 4D flow MRI can be used for detailed in-vivo assessment of vascular hemodynamics with 3D visualization and quantification of aortic flow. Advances in rapid prototyping (3D printing) have also provided the ability to manufacture complex models quickly and at low cost. The purpose of this study was to illustrate the feasibility of combining 4D flow MRI with 3D printing to create a comprehensive tool for cardiovascular evaluation.

## Methods

**Figure 1:**
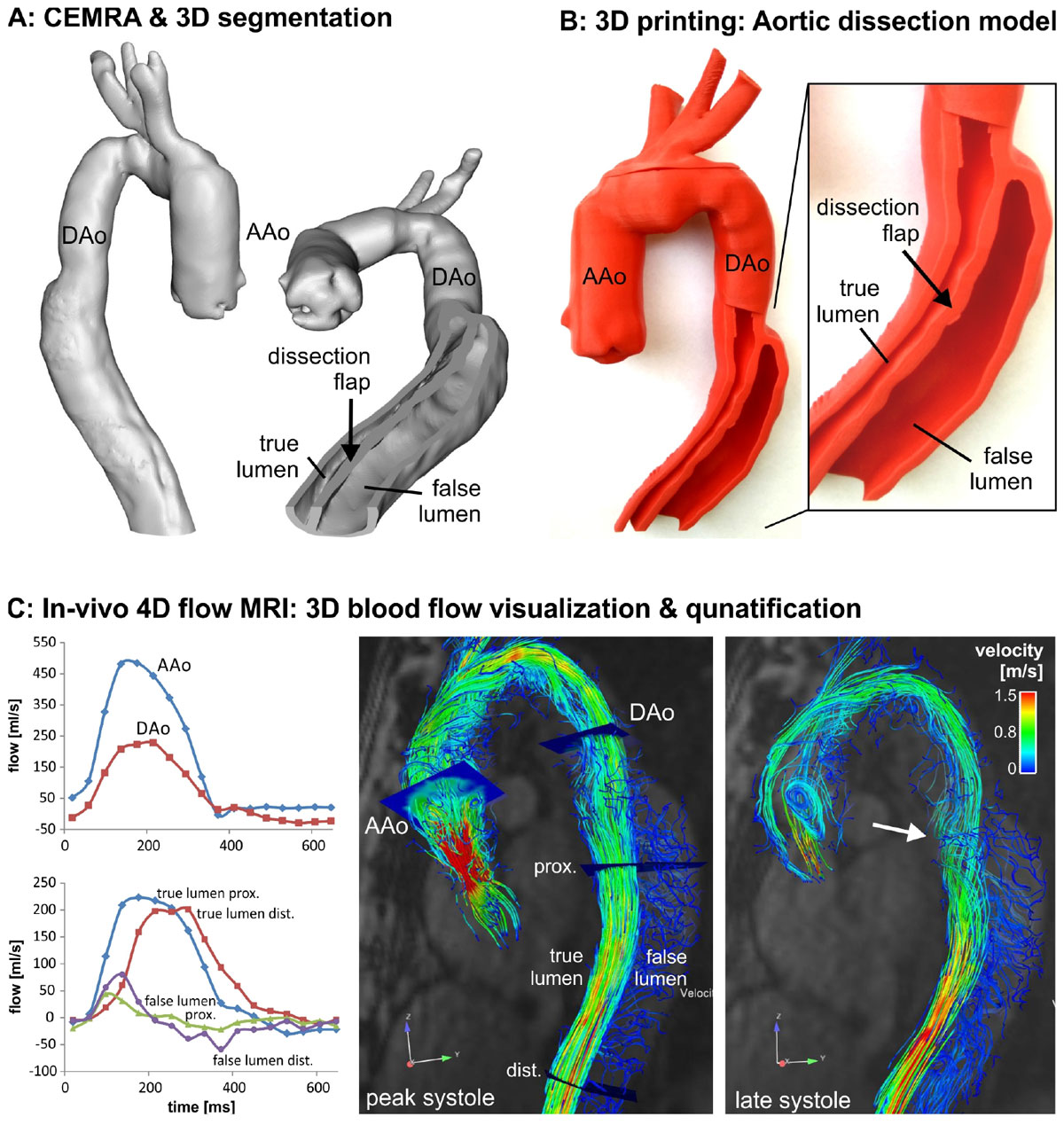
**Integration of 3D CEMRA based segmentation of the aorta (A), 3D printing of a one-to-one replica of the patient’s aorta and dissection geometry (B) and in-vivo 4D flow MRI for the evaluation of aortic 3D hemodynamics (C)**. The white arrow indicates complex helix flow pattern near the entry point into the true proximal lumen. AAo/Dao: ascending/descending aorta, prox: proximal, dist: distal.

We present the case of a 55 year old male (EF = 59%) with chronic descending aorta dissection. Contrast-enhanced MRA and 4D flow MRI were performed for in-vivo measurement of blood flow dynamics. Using CEMRA data in segmentation software (Mimics^®^, Materialise) a digital model of the aorta was generated (figure 1A). A 1:1 replica was 3D printed (1B) using Makerbot Replicator^®^ 2X (max build size 246 × 152 × 155 mm^3^, layer resolution 100µm). 4D flow analysis included phase offset error correction (Maxwell terms, eddy currents, velocity aliasing) with flow visualization using 3D streamlines. Quantification was performed using planes at mid-ascending aorta (AAo), proximal descending aorta (DAo), and at proximal and distal true/false lumens along the dissection (1C). The 3D print model and 4D flow findings were demonstrated to a volunteer group of radiologists and a Likert questionnaire was used to gauge their view on utility of the model in clinical practice.

## Results

Computer time for segmentation, modeling and 4D flow analysis was 90 minutes. 3D printing took 7 hours at a total cost of $15US. The dissection in the DAo, true/false lumens and dissection flap were clearly visible in the printed model. 4D flow analysis revealed high flow in the true lumen and substantially reduced flow in the false lumen during systole (Figure 1C). 3D visualization showed complex helical flow near the true lumen entry point (1C, white arrow). Quantification confirmed high flow (peak velocity 1.64 m/s) in the true lumen, whereas false lumen flow was markedly lower (peak velocity 0.43 m/s) with retrograde flow during diastole (1C, left). Clinician response to the questionnaire was overwhelmingly positive as summarized in Table 1.

**Table 1:** Clinician Survey Results

Inquiry	Strongly Agree (5)	Agree (4)	Neutral (3)	Disagree (2)	Strongly Disagree (1)	Mean	Std Deviation
(*) 3DP helped with visualization of this case	2 (22%)	7 (78%)	0	0	0	4.23	0.44
3DP can generally help visualize patient anatomy	3 (34%)	4 (44%)	2 (22%)	0	0	4.12	0.78
3DP + 4DFlow beneficial in my clinical practice	0	6 (67%)	3 (33%)	0	0	3.67	0.50
3DP helpful when discussing findings with colleagues	4 (44%)	5 (56%)	0	0	0	4.45	0.53
3DP helpful in educating students/residents	8 (89%)	1 (11%)	0	0	0	4.89	0.34
3DP helpful when discussing findings with patients	7 (78%)	2 (22%)	0	0	0	4.78	0.44
3DP can have value for intervention planning	1 (11%)	6 (67%)	1 (11%)	1 (11%)	0	4.78	0.83
I can see 3DP become important part of my practice	0	4 (44%)	5 (56%)	0	0	3.45	0.53
(†) 3DP time of 7-8 hrs for model is reasonable	1 (11%)	4 (44%)	1 (11%)	2 (23%)	1 (11%)	3.23	1.3
Expenditure of $15US for 3DP model is reasonable	4 (44%)	5 (56%)	0	0	0	4.45	0.53

3DP = 3D Printing

N = 9 (3 attending radiologists, 3 fellows, 2 residents, 1 medical student); all participants were male except one attending who was female

Average age 33.6, std = 4.6 yrs; None of the participants had any practical experience with 3DP

All values in Likert survey were standardized (Strongly Disagree = 1 to Strongly Agree = 5; N/A valued at Null). % are approximates.

(*) There was overwhelmingly positive response by clinicians to patient-specific 3D print model for the presented case.

(†) The area of highest clinician concern (length of time) also had widest spread (Std = 1.3)

## Conclusions

The results of our study demonstrate the feasibility of combining in-vivo MRI and 3D printing for the comprehensive evaluation of aortic abnormalities like dissection. Clinicians are enthusiastic about the prospects of 3D printing patient-specific anatomy thus larger studies seem warranted to assess the value-added potential for diagnostics, medical education, surgical planning, and communication of radiologic findings to patients. Combined with 4D flow MRI, 3D printing also enables in-vitro simulation of interventions like graft repair on aortic flow characteristics, opening new horizons in procedural planning.

